# How localized are computational templates? A machine learning approach

**DOI:** 10.1007/s11229-023-04057-x

**Published:** 2023-03-13

**Authors:** Maximilian Noichl

**Affiliations:** 1 Faculty of Philosophy and Education, University of Vienna, Universitätsstraße 7, 1010 Vienna, Austria; 2 Faculty for Social Sciences and Economics, University of Bamberg, Feldkirchenstraße 21, 96045 Bamberg, Germany

**Keywords:** Model templates, Computational templates, Modeling practice, Formulas, Science mapping, Digital humanities, Computational philosophy

## Abstract

A commonly held background assumption about the sciences is that they connect along borders characterized by ontological or explanatory relationships, usually given in the order of mathematics, physics, chemistry, biology, psychology, and the social sciences. Interdisciplinary work, in this picture, arises in the connecting regions of adjacent disciplines. Philosophical research into interdisciplinary model transfer has increasingly complicated this picture by highlighting additional connections orthogonal to it. But most of these works have been done through case studies, which due to their strong focus struggle to provide foundations for claims about large-scale relations between multiple scientific disciplines. As a supplement, in this contribution, we propose to philosophers of science the use of modern science mapping techniques to trace connections between modeling techniques in large literature samples. We explain in detail how these techniques work, and apply them to a large, contemporary, and multidisciplinary data set (n=383.961 articles). Through the comparison of textual to mathematical representations, we suggest formulaic structures that are particularly common among different disciplines and produce first results indicating the general strength and commonality of such relationships.

## Introduction

How are the sciences organized? Given that individual disciplines do not just stand next to each other in an unrelated fashion, but have overlapping objects of inquiry, and often borrow methods from their neighbors, it seems sensible to ask what organization arises among them. One view that has been historically prominent, is that the sciences are organized in a somewhat hierarchical order, which leads from physics over chemistry to biology, and from biology and neurology on to psychology and the social sciences.^1^ The organization principle along this gradient is frequently characterized by growing complexity, by ontological relationships (e.g. of composition), or by explanatory relationships. Interdisciplinary work, under this view, tends to occur at the borders of connected areas, whose objects of study blend into each other. Other common views might highlight a contrast between more theoretical and practical fields,^2^ in which more applied fields connect to mathematics, and the more theoretical areas of computer science and physics.

Philosophers of science have further complicated this picture, by highlighting connections between scientific disciplines orthogonal to these arrangements.^3^ In particular, the recognition of interdisciplinary similarities of models, which can arise through model migration or the convergent evolution of modeling practices, has shifted our image of what factors enable disciplinary contacts. Philosophers have analyzed these connections through notions such as computational templates,^4^ model templates^5^ and theoretical templates,^6^ citing the Lotka-Volterra-, the Kuramoto-, and the Ising-model, various commonly used statistical distributions, and generative network models as primary examples. Examples that, both in the scientific literature, as well as in the philosophical literature referencing it, are commonly introduced by one central mathematical formula.

The analyses of these examples have greatly contributed to our understanding of interdisciplinarity and model transfer. But due to their focus on specific cases, there are certain limits placed on how much they can tell us about the extent to which the underlying mechanisms play a role in the sciences seen as a whole. The investigated cases might for example turn out to be rare episodes uncharacteristic of the common conduct of scientists in a domain, or they might attach themselves to views or personalities that turn out to be at the very fringes of their disciplines. Demonstrating that one template is in use in very different places, even though it might appear there under very different names, and with little attribution to earlier occurrences in other disciplines, is challenging on its own.^7^ It seems even harder to show that this is a phenomenon that is substantial enough to be considered a structuring principle of the sciences.

Large-scale questions like this, asked about an ever-growing scientific landscape,^8^ as well as new technological possibilities and the increasing availability of data-sets, have recently led some philosophers to embrace the use of digital methods.^9^ These have become attractive to researchers, as they allow engagement with vast amounts of material that due to their scale are inaccessible to traditional methods.

In this contribution we will make use of such novel computational techniques, drawing on the science mapping literature, to provide a first bird’s eye view of what the answer to this question might look like. After a brief overview of how techniques of science mapping have become adopted, we will describe a technique that allows us to find connections between the mathematical apparatus of articles, by calculating the similarities of formulas. We will apply this technique to a sample of 383.961 preprints drawn from various disciplines, and through the comparison of textual to mathematical maps suggest formulaic structures that link disciplines, as well as those that are particular to specific disciplines.

## Science mapping

Categorizations of science and of the knowledge it produces, have a long history in philosophy. Some philosophers, like Hobbes^10^ or the *Encyclop*é*distes*^11^ also chose to visualize their classification schemes in tree-like structures, a visual history which Weingart (2013b) traces back to Porphyrian trees, and expands in a modern shift towards web-like models of science.^12^

With the introduction of digital databases of scientific output and the development of the powerful computational resources needed to process them, new approaches to the structural mapping^13^ of the sciences have become possible. Their attraction lies in their ’data-drivenness’, which means that the structures they reveal are thought to be determined only by the available material and the method of processing. And as the method of processing is usually not domain-specific, so the thought goes, they allow the connections to show themselves relatively removed from the structures imposed on them by institutions and classification systems.

There are several data types commonly used in the production of science maps. Most commonly, we see mappings that make use of some type of citation data, either establishing links between articles if one article cites another, if two articles are cited by the same text (co-citation networks), or if they cite the same text (bibliographic coupling).^14^ The other common data source for mappings of science are texts themselves, which can be arranged by their semantic content.

**Fig. 1:**
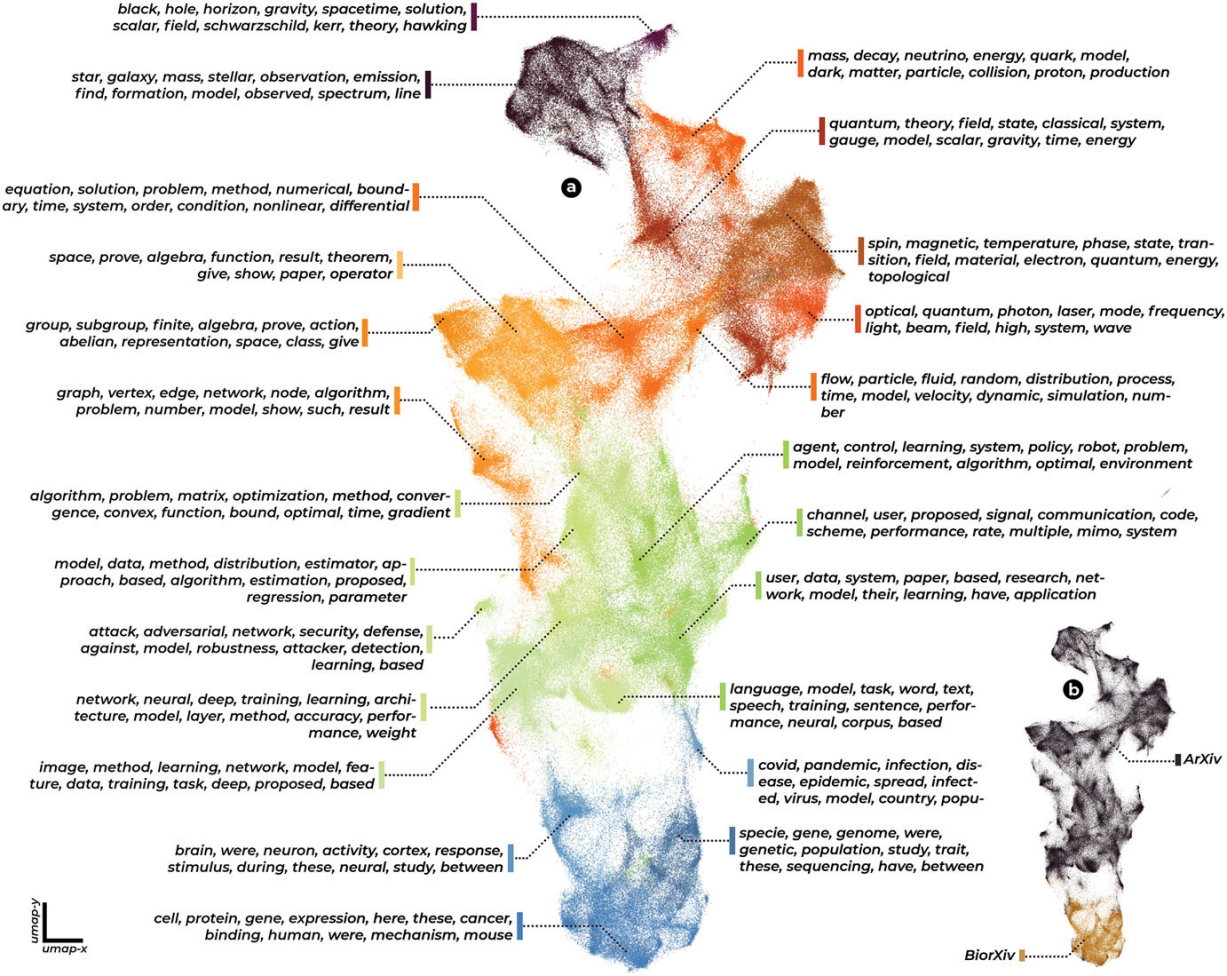
A science map, based on all preprints archived in the arXiv and the bioRxiv, in the years 2019 and 2020. The mapping suggests a gradient from physics, via mathematics and computer science to the life sciences. We also observe, on the lower right, a relatively clear separation between the arXiv and the bioRxiv. We consciously avoid reifying clusters by assigning labels to them. Instead, we indicate the keywords that achieve the highest summed-up tf-idf scores for each cluster, indicating the words that are most specific to each cluster, as opposed to the rest of the sample. This and all following plots were produced in matplotlib, and typeset using Adobe InDesign

We present such a map in Fig. 1. We will go into much more detail about how it was made, and how we should interpret it, in the next section. For the moment it suffices to say that it consists of 383.961 recent preprints, drawn from physics, mathematics, computer science, and the life sciences, and arranged by an algorithm that was unaware of their origin. Clusters in the mapping are labeled by their most distinct keywords. It is not hard to read the involved disciplines from them. Towards the top of the graphic, we note various sub-disciplines of physics (dark violet, red), moving down into mathematics (orange), computer science (light green), and finally at the bottom, the life sciences (blue).^15^

Because of the peculiarity of the data source (it lacks chemistry, and therefore fails to establish links between physics and biology, it also lacks most social sciences), we shouldn’t make any too involved claims about the global structure of science as a whole from this map, although it would seem to fit reasonably well into the consensus reported in the literature.^16^ But we can point out that locally, the arrangement and connectivity of the clusters make a lot of sense. The cluster of neurology at the bottom left for example fades upwards into the computer science cluster associated with the training of neural networks. The, at the time of sampling, just emerging cluster of Covid-19-literature, takes up space between the life sciences and computer science as well, owing to the computerized nature of modeling and prediction during the early pandemic. Towards the middle left we see an elongated cluster of distinct network science traveling down in parallel, with several connections to areas in mathematics and computer science. All in all, the picture seems to correspond relatively well to a view of the sciences as thematically separated units, which are partially linked by interdisciplinary endeavors. We further note a kind of theoretical ’backbone’ leading from theoretical physics, over some areas of mathematics, to parts of computer science, suggesting a connection of theoretical fields, from which more application-centered ones radiate.

The thematic map, therefore, seems to encapsulate many of the common-sense assumptions about the structure of science, which we had identified above. We can now ask how it compares to a picture that brings the connectivity introduced by mathematical methods to the forefront. But first, we need to get into some technical details about how these maps are produced, and what we need to keep in mind when reading them.

## Sample description

In the present contribution, we draw our sample from two large preprint-repositories, the well-established arXiv, which mainly contains material from physics, informatics, mathematics, and the younger bioRxiv, which is focused on the life sciences. Preprints are by now a very common form of scholarly communication in many (although not all) scientific disciplines, both to scientific peers and the general public,^17^ which would make them of interest to philosophers of science, even if it weren’t true that a sizeable share of them later do become regular journal articles.^18^ While preprints certainly do not form a perfect mirror image of the scientific literature in their respective domains, they can still be considered a reasonably close proxy. In our case, we focus on preprints, because they are commonly archived in large databases of relatively uniform format, which is not the case for published material. We are in particular need of a uniform format because, without it, the reliable parsing of formulas becomes an exceedingly difficult problem. This is also the reason why, in this contribution, chemistry and the social sciences have been left out. While chemists have recently begun to establish a central, uniformly formatted preprint-archive, ChemRxiv, it has not been as well adopted at the time of writing, as the ones included in our sample, and can’t be considered as representative. And while some social sciences have strong preprint cultures, they tend to archive their preprints solely in pdf format, with source files remaining inaccessible.

Because we are nonetheless interested in interdisciplinary relationships and attempt to construct a large, contemporary, yet consolidated multidisciplinary sample, we settled on all preprints archived in the arXiv and the bioRxiv in the years 2019 and 2020, which makes for a total of 383.961 articles, 49.769 of which stem from the bioRxiv, and 334.192 from the arXiv. Full preprints and associated data were downloaded using the available AWS-services^19^ maintained by the respective providers. Metadata for the BioRxiv was downloaded using the provided API, metadata for the arXiv was downloaded from the Google-Bucket provided by Cornell University (2020) and *kaggle*.^20^

## Processing

**Fig. 2:**
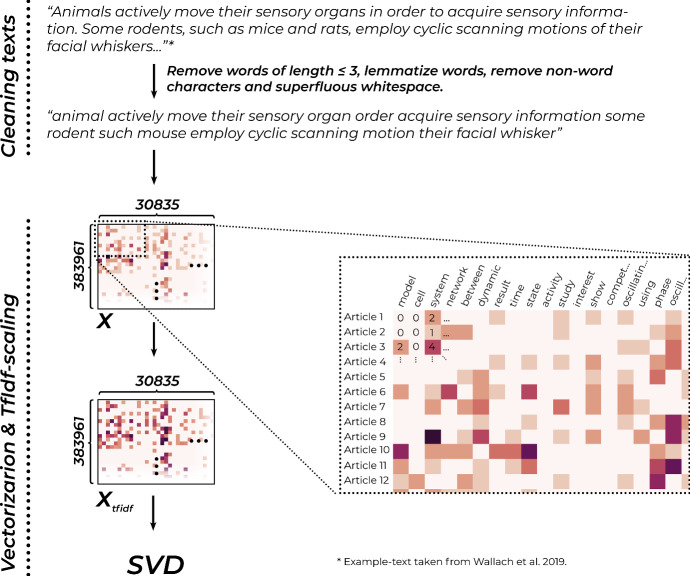
The vectorization process, illustrated. For each text we count how often each word present in the vocabulary appears, building a huge word-frequency table. We then adjust these counts using a tf-idf-scheme, which increases the impact of less common words

### Constructing the thematic mapping

To construct the thematic mapping, we proceed in a mode of computational text analysis that has become fairly common in recent years.^21^

The first step of the process is illustrated in Fig. 2. We first clean the texts by removing words with less or equal to three letters,^22^ non-word characters, and superfluous white space. We then lemmatize words,^23^ which means that inflected words are moved into a uniform base form, so that e. g. ‘animals’ becomes ‘animal’ and ‘playing’ becomes ‘play.’ We then transform the texts into so-called *bag-of-words*-vectors. This means that we construct a very large table (383961 rows * 30835 columns, implemented as a scipy-sparse matrix^24^ in which each row represents one of the articles in our sample, and each column represents a word.^25^ Each cell in the table contains how often each word occurs in the respective article. This approach is called bag-of-words, because it neglects all internal structures of the texts, and reduces them to distributions of word frequencies. We choose this simple, yet remarkably effective approach, because it makes our later analyses straightforwardly interpretable, as well as easier to troubleshoot.

Most cells contain the number zero, as most words do not occur in most articles. We assume that very common, general words, like ‘model’, tell us rather little about the thematic structure that we are interested in. But because they are so common, they might exert a strong influence on our results. For this reason, we first remove common stopwords, like ‘they’, ‘them’ or ‘and’,^26^ and then re-scale the counts in a way that increases the weight of very uncommon words, while decreasing that of common words, using what is called a tf-idf-weighting-scheme. To conduct tf-idf (*Term frequency - inverse document frequency*), we first calculate a weight for each word. To do this we divide the number of documents by the number of documents in which the word of interest appears, resulting in a number that will be far larger for rare words than for frequent ones. We then take the logarithm of this number, to moderate its effect, before we multiply it with every word count in the column that is associated with the word. This increases the influence of infrequent words, which are more useful for differentiating between texts while decreasing that of very frequent ones.

**Fig. 3:**
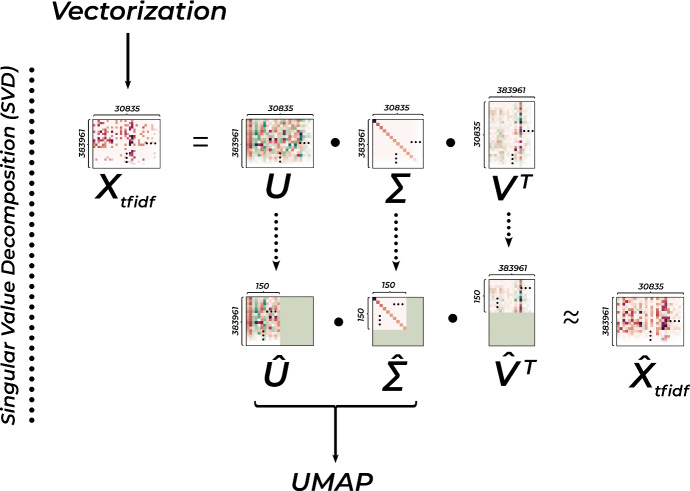
In SVD, the initial data-matrix $X_{tfidf}$ gets decomposed into three constituents - *U* which contains the Eigenvectors of the row-wise covariance-matrix, $V^T$ which contains those of the column-wise covariance-matrix, and $\Sigma$ which contains the Eigenvalues. When multiplicated together, these matrices result in $X_{tfidf}$. (We can’t go into the exact details of *how* this decomposition is accomplished computationally.) If we truncate them though, so that e. g. only the first 200 columns of *U*, the first 200 rows of $V^T$, and the upper 200 * 200 corner of $\Sigma$ remain and multiply the resulting matrices $\hat{U},\hat{V^T},\hat{\Sigma }$, we get as a result an approximation of the original matrix. Indeed we notice that $\hat{X}_{tfidf}$ in the lower-left exhibits nearly the same patterns as $X_{tfidf}$, having its values only slightly jittered. As we are only interested in recovering the row-wise correlations with the major axes in the dataset, we can directly go on to conduct our calculations with the small matrices $\hat{U}~\varvec{\cdot }~\hat{\Sigma }$. For further introduction to the process, see Petrovich (2020)

The resulting table of tf-idf-scores then gets passed to a technique called *Singular Value Decomposition*. SVD is a general method that decomposes one matrix into three new ones, which, when multiplied with each other, result in the initial one. These matrices can be interpreted as the Eigenvectors of the covariance matrices of the rows and columns, and their Eigenvalues. This means that they encode how much individual data points co-vary with the dominant axes of co-variance, or in other words, how much they agree with the most dominant trends in our dataset. A more detailed explanation of this technique is given in Fig. 3.

By multiplying together only the parts of these matrices, which are responsible for the 150 most dominant features, we can achieve an approximation of the initial matrix, which keeps intact its most important features, while resulting in a much smaller dataset. The reason for conducting this intermediate step is twofold: on the one hand, it decreases computation times downstream by making the dataset smaller. On the other hand, it reduces noise in the dataset, as the resulting matrix keeps the largest axes of variance in the dataset intact, but clears out smaller ones.

In the final step towards our mapping, we conduct *Uniform Manifold Approximation and Projection* (McInnes et al. 2018) on the SVD-vectors. UMAP is a relatively young dimensionality-reduction and visualization technique, which has quickly risen to popularity in a wide range of analysis tasks.^27^ While it can play a variety of roles in processing pipelines, its current main application is to give a two-dimensional representation on a very high-dimensional dataset.

It should at this point be clearly stated, that it is impossible to perfectly fulfill this task in the case of most natural datasets - 150 dimensions just do not fit into two.^28^ But in many cases of natural datasets, the sub-processes in the generation of the dataset in which we are most interested, take place in a space of a dimensionality that is lower than that of the raw dataset.

We can understand this by using our textual dataset as an example. As we recall, it springs from two largely non-overlapping sources: Texts drawn from the life sciences, and texts drawn mainly from physics and informatics. So while there is of course much room for overlap, we expect this split to be reflected in our dataset in some major way. This is one important aspect of the data-generation-process, that has no trouble fitting into two dimensions, even though the actual dataset if prepared through the bag-of-words method, will be of far higher dimensionality. And indeed, if we look at our textual map in Fig. 1, colored by the source of the articles, we find a relatively clear arrangement along the y-axis. So while remaining necessarily unsatisfactory to some degree, we can expect to find global structures represented in our mappings, which, if interpreted with adequate care, can yield deep insights into our dataset.

**Fig. 4:**
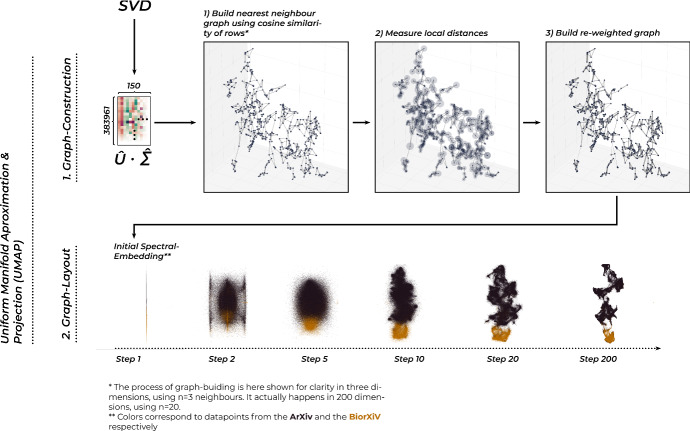
A graphical explanation of UMAP. We begin by constructing a graph that links each data point to *n* of its nearest neighbors. We then reweigh the edges of the graph and lay them out in the low-dimensional space using a force-directed algorithm. Graphic inspired by Lee et al. (2021)

With these cautionary remarks out of the way, we can now proceed to give a rough explanation of how the algorithm works. The process is also visualized in Fig. 4. It begins by constructing a nearest neighbor graph from our dataset, in which each data point is linked to a predefined number of closest neighbors. The ‘nearness’ of data points is determined by calculating the *cosine-similarity* between the rows in the dataset, a measure that is usually recommended for textual data because it weights the individual features similarly. If we interpret the rows of our working dataset as vectors that determine positions in a vector space, we can understand cosine similarity as the angle between these vectors in the origin. The weight of the edges in this graph is determined by the similarities, and it is what will later co-determine how near or far from each other points are placed in our mapping.

The issue we now run in, which is commonly known as one instance of the ‘curse of dimensionality’, is that in this high-dimensional space, most distances appear to be nearly identical, which means that the weights are not very informative as they are. For this reason, UMAP employs a reweighing scheme that adjusts weights based on local distance measures at each node of our graph.

Having conducted this reweighing step, we now have to find a satisfying layout for the graph. UMAP generally starts by conducting a quick spectral embedding (a rough first layout) of the graph and assigning the resulting coordinates as initial positions to the data points. From there it follows the common idea of many network-layout algorithms, which is to have a general repulsive force, which pushes all nodes of the network constantly away from each other while using the weights as links that pull them together at the same time. As shown in Fig. 4, simulating the interplay of these two forces on the nodes moved by them, yields after a few hundred simulation steps an approximation of the initial configuration of similarities of points in our low-dimensional, perceivable data space.

**Fig. 5:**
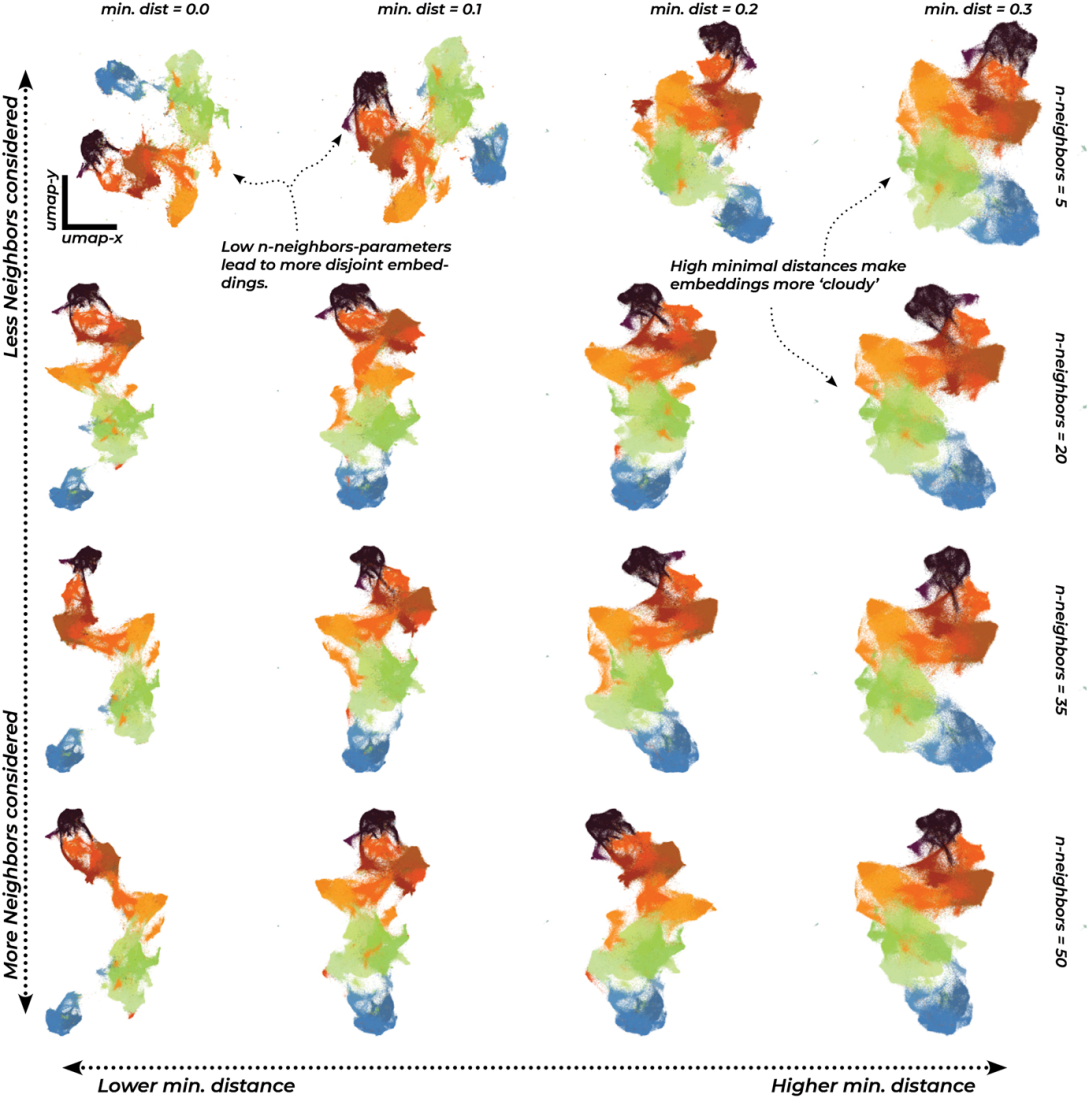
Results of different runs of the UMAP algorithm, demonstrating a range of possible outcomes under different hyper-parameter settings. We note how some features of the embedding, e.g. the left-right orientation are contingent, while the global structure stays relatively robust, except under very small values of the n-nearest neighbors parameter

We should remind the reader at this point again about the noted imperfections of the resulting mappings. To give an example of one common issue that can arise due to the construction of the nearest neighbor graph: If a data point were to be completely disconnected because it was just so far away from all the other points that it couldn’t be included in any other points’ nearest neighbors, the algorithm would have no idea where to place it, and it would fluctuate just randomly around (and away from the other points) during the layout process. This, and similar problems, don’t necessarily endanger our purpose. But it is good to keep in mind, that not all distances, especially between individual points instead of larger groupings, can be expected to be interpretable. Instead, it makes more sense to visualize a whole range of possible embeddings, as we do in Fig. 5, to see which features of our embedding remain consistent over changing hyper-parameters and the uncertainties of the stochastic layout processes.

An additional sanity check here is to conduct clustering using a graph clustering algorithm^29^ on the nearest neighbor graph underlying the embedding. As the clustering solution is independent of the layout process, it can flag mismatches for us, and provide insights into the nature of the structures that arise in the UMAP mapping.

We now turn to the analogous construction of the map of mathematical content.

### Constructing the mathematical mapping

In constructing our map of the mathematical content, we will try to keep as close to the way we constructed our thematic map as possible. But calculating the similarities between equations is not trivial, and is a far less studied problem than that between texts. A simple bag-of-words approach as outlined above for example must fail because the number of individual symbols used in mathematics is much smaller than the number of words that can be found in a natural language corpus.

**Fig. 6:**
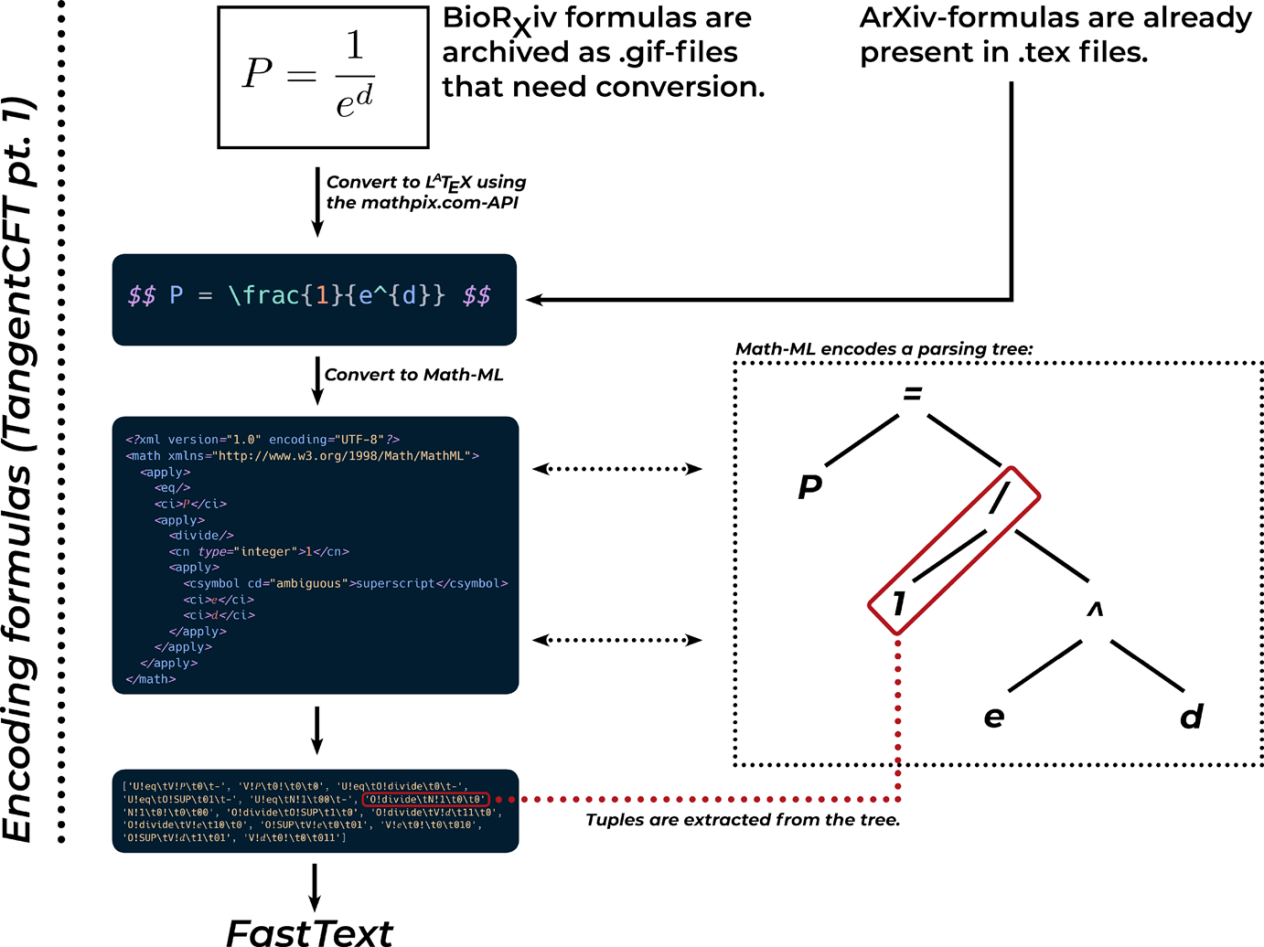
The formula-preprocessing process inspired by Tangent (Zanibbi et al. 2016), using the implementation from TangentCFT (Mansouri et al. 2019)

Even worse, often some mathematical structure might be filled with different symbols or names for variables, even though the same calculation is conducted, as seems to be often the case with $p,~q$ and $\phi ,~\rho$ respectively. But an approach that skips all symbols in favor of pure surface structures will be insufficient as well, as mathematic content does not determine specific surface structures: the same calculation can be described through formulas, that on the surface level seem to be quite different. The straightforward operation of the mean, for example, can look like $\frac{m_1 +... + m_N}{N}$, as well as $\frac{1}{N} \sum _{i}^{N} m_i$. But just counting the co-occurrences of specific symbols between formulas, in the case of the mean e. g. $m,~N,~\frac{...}{...}$ doesn’t suffice either, as the individual symbols have semantics that in themselves are quite complicated and multifaceted. The letter $n$, for example, tends to take on a very specific meaning, denoting the size of a (sub-)group, which only rarely will be replaced by other letters in that position. Similar things are true for $x$, $y$, and $z$, which commonly, but not always, are used to indicate spatial coordinates. The letter $i$ might interact with $n$ as a counting variable, but on its own will more often denote the imaginary unit. And the letter $e$ can rarely be replaced by the preceding $d$ without a complete change in meaning. This problem is even more challenging, as the same is not true for all letters and symbols, some of which have far more free or diffuse semantics.

For these reasons, our similarity score can’t depend on a simple idea of how often a certain letter or symbol is present in the two formulas whose similarity is to be determined. It has to be aware of the *context* in which a certain symbol presents itself. It has to understand that e.g. a $\Sigma$ and a ‘+’ might in some contexts encode similar ideas, and totally unrelated ones in others.

We find an implementation of an approach that takes this into account in TangentCFT, introduced by Mansouri et al. (2019), to whom our contribution is very much indebted. Their basic insight is that it is more promising to encode formulas not through individual symbols or pure structures, but through a combination of those ideas: A list of structure-encoding symbol-tuples which are then fed to a language model that is able to learn context-depending representations.

The general process is described in Fig. 6. After all formulas have been brought into a uniform^30^ LaTeX-format, they are transformed to Math-ML, which encodes them in a hierarchical structure, in which each node gets linked to the one directly above. In the term $\frac{1}{e^{d}}$, for example, the symbols $e$, $d$ and the superscript relation between them all get linked to the fraction in the denominator position, while $1$ gets linked to the numerator. Each of these linkages along the tree can be considered as a tuple of a relation and a symbol, which can be used in further modeling.

This representation scheme was initially proposed by Zanibbi et al. (2016) with the idea to build a search engine for formulas. Their idea was that formulas that shared large parts of these graphs could sensibly be considered similar, and would then be returned by the search engine. While this had shown some promise, this approach still struggled with the complexities and indeterminacies of the mathematical language, which we have outlined above.

For this reason, more recent approaches have turned to *embedding* the hierarchical formula representations. This means that instead of just counting the tuples, we try to learn representations that encode the similarities between them and make these useful for the representation of whole formulas. Mansouri et al. (2019) use the *FastText*-architecture for this purpose. FastText was initially developed by Facebooks AI research-lab as a technique for word embedding, with the intention of text-classification.^31^ In the presented version of the technique, we use tuples, where in the standard use-case one would use words.

**Fig. 7:**
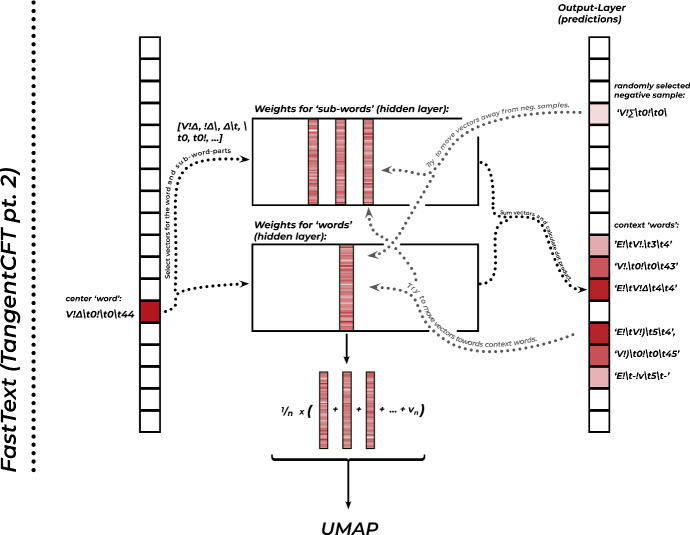
A simplified illustration of the training of the FastText-model. The one-hot encoded vectors, on the left, which represent one ’word’ in the whole vocabulary, select its representation in the hidden layer, as well as that of its sub-word parts. The sum of these vectors is then used to form a positive prediction on the context of the word, and a negative prediction on randomly selected, unrelated words. The prediction errors are then used to adjust the weights in the hidden layer. In the end, we use those internal weights to represent our formulas in further processing

Very roughly explained, FastText ’learns’ the semantic relations between words, from the contexts in which individual words occur.^32^ For this, it cuts up all words into chunks of three letters, for which it will learn representations as well. This way it can later also encode unknown, rare, or inflected words if they contain already known sub-word parts.^33^ At the beginning of the training process, all representations are just random strings of numbers, without any informational content. The algorithm now repeatedly - many hundred-thousand times - takes chunks of text of a certain size (the window size, which we set in our model to a value of 13), then selects the word in the middle of the chunk as the target word, while the surrounding words are kept as context words. It looks up the representations of all the sub-word representations of this word and sums them together with the word representation itself. It then retrieves all the representations that we have gotten so far for the context words, as well as some additional randomly chosen ’contrast’ words (in our case 15 of them). Through a process called stochastic gradient descent, it then ’jitters’ the representations of the context words in a way that makes them more similar to that of the target word, while at the same time moving them away from those of the randomly chosen contrasts. We illustrate this feedback process called backpropagation in Fig. 7. In each step, the representations are changed only a little bit. But over many training steps, the representations begin to encode the relations between words remarkably well. This process opens up several applications. Using the finished representations, we can find synonyms in the corpus by searching for the words most similar to a query word, predict missing words from a text, or, as we will see now, judge how similar two texts are, based on the representations of the words they contain.^34^

At this point though, in our application, we are not interested in words. Instead, we apply the FastText algorithm to the tuples that we built earlier from the parsing trees of formulas. These tuples arise from the connections of particles of equations along the order of operations, meaning that they can encode both structural properties and differences between symbols. In this way, we find representations of the particles of formulas that encode relationships like the similarity between $\frac{1}{n} * m$ and $\frac{1}{n} * k$, but can encode also the difference between ’i =’ in the formula $\sum _{i=1}^{n} i$ and ’i squared’ in the formula $i^{2} = -1$. But what to do now with these encoded tuples? Recall from earlier, that the representations of the tuples which we have learned through FastText are simply long columns of numbers. So, to get back to formulas, for each formula we simply select the representations of all the tuples that are present in it, and take the mean along one axis, so that the result is a new representation that is influenced by the representations of all the tuples present in the formula. Formulas that contain the same, or very similar elements, will end up with more similar aggregated representations, while formulas that have little in common on the tuple level, will end up with very dissimilar ones.

Concretely, we extract from the collected arXiv preprints all LaTeX equation environments and math modes that are longer than 15 and shorter than 800 letters. The underlying idea is, that we largely want to avoid formulas that are just tiny particles, or value assignments (expressions like $n=30$), as well as certain misappropriations of the formula environment, e. g. when authors use it to typeset longer pieces of texts. In the case of the bioRxiv, formulas are presented not in LaTeX  originally, but we have to convert them from images to a uniform LaTeX-format using the webservice mathpix.com. In this case, we limit ourselves to images that are smaller than $800 \times 100$ and larger than $200 \times 40$ pixels. We also limit in both cases the number of formulas per article to a maximum of 20 randomly selected ones out of all eligible ones, to avoid very formula-rich articles overpowering the others with their influence. On the whole, this leaves us with 1.691.372 formulas, from which 1.656.929 originate from the arXiv, and 34.443 from the bioRxiv. All of these formulas are used in the training process of the model, but because of computational limitations, only 500.000 randomly selected formulas are used in the analysis.

**Table 1:** Formulas in our dataset, which our model considers most similar to a common expression of the normal-distribution: $f(x)= \frac{1}{\sigma {\sqrt{2\pi }}} e^{-{\frac{1}{2}}{\frac{x-\mu }{\sigma }}^{2}}$, in decreasing order of similarity. We note that while the most similar results at the top are virtually identical, less similar ones towards the bottom switch out individual letters, or modify the formula with additional terms

Result	Dist. to query
$f(i)= \frac{1}{\sigma \sqrt{2\pi }} e^{-\frac{1}{2}(\frac{i-\mu }{\sigma })^2}$	0.001
$f(x)={\frac{1}{\sigma {\sqrt{2\pi }}}}e^{-{\frac{1}{2}}\left( {\frac{x-\mu }{\sigma }}\right) ^{2}}$	0.01
$g(\epsilon )=\frac{1}{\sqrt{2\pi }\sigma }e^{-\epsilon ^2/2\sigma ^2}$	0.011
$w_x = \frac{1}{{\sigma \sqrt{2\pi } }} e^{{{ - \left( {\eta _x(B) - \mu } \right) ^2 } / {2\sigma ^2 }}}$	0.012
$P(x) = \frac{1}{{\sigma \sqrt{2\pi } }}~e^{{{ - \left( {x - \mu } \right) ^2 }/{2\sigma ^2 }}}$	0.013

**Fig. 8:**
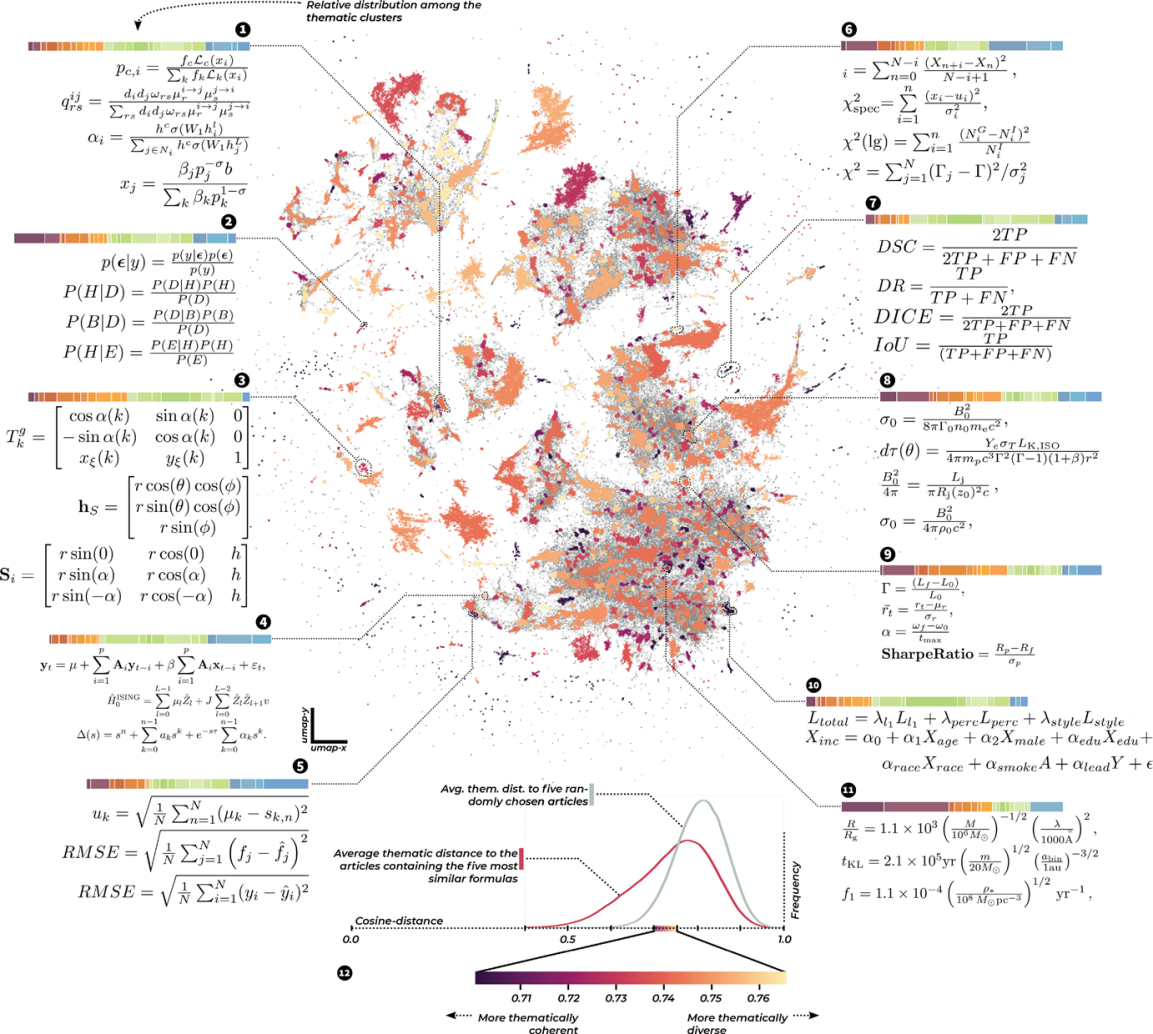
A mapping of 500.000 formulas by their similarity. A few clusters are annotated for illustration purposes with example formulas that were selected from the formulas closest to the cluster centers. We note several well-known patterns, such as, among others, Bayes’ theorem (2), $\chi ^2$-statistics (6), and the Root Mean Squared Error (5). We show the thematic composition of the articles from which the formulas in each cluster originate in color bars, using the same colors as in Fig. 1. In (12) we show the overall distribution of average thematic distances between the article from which each formula originates, and the articles of origin for the five closest articles, and compare it to a random selection of articles. This measure suggests that while there clearly is some thematic structure to the distribution of formulas - as evidenced e. g. in (7, 10, 11) - the broad distribution of mathematical forms is the rule, not the exception

Moving forward in our analysis, after producing representations for each of our tuples, we can use them to build representations of our formulas by taking the representations of each tuple and taking the mean of them, yielding one single new formula vector. We can check whether this works as well as in Mansouri et al. (2019), by querying example formulas to the resulting model, and see, which formulas the model suggests are most similar to them. The result of one such test is reported in Table 1. And in the same way, in which we laid out the articles by their semantic similarity using UMAP, we can lay out the formulas, after calculating the similarities between them, using UMAP. The resulting formula map is reproduced in Fig. 8.

We should note here, that the notion of similarity between formulas, which our measure encapsulates is not necessarily one of deeper mathematical connections. The formulas that UMAP groups together into one cluster, will not necessarily all have a single structure in common. Rather they will have multiple overlapping similarities. In this application, we switch due to the large sample size to another clustering algorithm, hDBSCAN,^35^ which is commonly suggested to be paired with UMAP.

A quick glance at the mapping of formulas shows it to be much more disjoint than the mapping of articles. To get some insights into what each formula cluster contains, we have selected example formulas for some of them and arranged them in boxes around the graphic. The example formulas were selected by calculating the mean of all formula vectors which were assigned to the cluster in question, and then selecting the formulas closest to the mean, which can thus be considered the primary examples from their clusters. We can easily make out versions of some very commonly used formulas, e.g. Bayes’ theorem, the root mean squared error, and the Sørensen-Dice coefficient, or formulas reminiscent of the Ising model.

We now have everything in place to return to our initial question: How does the thematic map of science, which corresponds to the common-sense picture of scientific organization, correspond to the structure encoded in the distributions of formulas? To analyze this correspondence, we have to check how thematically similar the articles are, which are held together by each cluster of formulas. We can do so by going back to the level of nearest neighbor graphs. For each formula in the embedding, we look up its 5 nearest neighbors, or in other words, the 5 most similar formulas, which are likely to be close in the UMAP-mapping.^36^ For each of these formulas we then look up the SVD-vector corresponding to the paper from which the formula was drawn and calculate the average thematic similarity of our original paper to the others. The resulting score indicates for each formula, how thematically similar the papers are, from which the surrounding formulas are drawn. The map in Fig. 8 is colored by these scores.

Now, if the distribution of mathematical methods were very specific to subject areas, the formula map would exhibit very low distance scores. However, this is not what we observe. While the thematic distances among formulas in our sample are clearly smaller than among randomly sampled ones, the difference is not drastic, and high thematic coherence seems to be mostly restricted to several small islands. The whole graphic generally indicates a relatively high thematic dissimilarity of formulas that are close to each other. Or in other words: The structure we have gathered from the formulas does not reconstruct the thematic picture of science.

## Conclusion

In closing, we ought to mention some limitations to this preliminary work. The first obvious limitation is that our sample, while rather large, does not include all areas of science that would be of interest. Not only are we missing chemistry, but also most parts of the social sciences, and works in the humanities that make use of formal methods. Another important limitation of this current approach is that the focus on formulas does miss out on the usage of models that are not explicitly introduced. This might especially confuse our picture of the parts of science that are heavily dependent on central software packages, which are already well understood by the respective scientific communities, and thus need no formal introduction. We see many opportunities for further work in this area that has to our knowledge not yet been adequately explored by philosophers of science. A final limitation of this contribution is the focus on exploratory methods. While we think there is good reason to think that the structures gathered through the proposed science-mapping approaches are informative of actual structures present in the sciences, they remain visualization methods providing orientation and background knowledge. It is at this point still an open methodological question how they can be linked to more rigorous statistical tests of hypotheses - although it might be also unclear whether this would increase their usefulness to philosophers of science, instead of moving the nature of the results firmly into the realm of scientometrics. Nonetheless, we would certainly like to see an uptake of the presented approach in the scientometric literature. While data with formulas in usable formats is certainly harder to procure for most areas than citations or full texts, we believe that additional, even larger-scale work might yield very interesting results here. Importantly, mathematical structures might not only be useful as a contrast-case to texts (or citations) but might be used in conjunction to build richer representations that do justice to complex phenomena of scientific transfer, such as model templates.

In our initial motivation for the use of computational techniques in philosophy of science, we suggested that the massive scale of contemporary science complicates case study approaches to philosophy of science. In view of our present investigation, we would thus suggest that it can be helpful for case studies, which clearly are necessary for any deeper understanding of interdisciplinary exchange, to be embedded into larger-scale computational analyses, which help to evaluate the likelihood that their results generalize. We imagine the relationship here as one of methodological triangulation, in which a mismatch between the results of the two approaches can serve as an invitation to reevaluate each one of them.

To summarize: We have asked how the common sense picture of scientific organization, corresponds to a picture drawn from similarities in the application of mathematics. To answer this question, we have introduced a new form of science mapping, and have presented its results when applied to a large, contemporary sample of scientific preprints. We have observed that even when taking a rather global view, the structure of the usage of mathematics in science is largely dissolved from its thematic structure. This suggests that the interdisciplinary similarities of models that have been observed by philosophers are by no means a niche phenomenon, or a mere curiosity, but represent a central organizational feature of contemporary science.

## Data Availability

The automated process for gathering the data presented in this article is made available under https://github.com/MNoichl/comp_templates. The aggregated and cleaned final datasets can also be accessed via this cod0e. The full code necessary for the replication of all results in this article is made available under https://github.com/MNoichl/comp_templates. The graphics in this paper were created in Python using matplotlib and seaborn, and assembled using Adobe InDesign.

## Footnotes

1 Auguste Comte (Comte and Martineau 2009) is commonly identified as the ideas modern originator, c.f. (Bourdeau 2022; Cole 1983). See also Arsenault et al. (2006), Fanelli and Glänzel (2013), Fanelli (2010), for empirical evidence for the hierarchies reality.

2 Peirce, for example, varies Comte’s classification by separating science of discovery, of review, and practical science. See Midtgarden (2020) for an overview.

3 See Humphreys (2002, p. 5), from whom the present investigation takes its starting point.

4 Humphreys (2002, 2004).

5 Knuuttila et al. (2007), Knuuttila and Loettgers (2014, 2016).

6 Humphreys (2019).

7 For a general account of how it can be challenging to derive theoretical conclusions from case studies in philosophy of science, see Kinzel (2016).

8 See Barnett and Doubleda (2020), Bornmann and Mutz (2015), Fortunato et al. (2018), Larsen and von Ins (2010) for estimates of the growth rates of modern science.

9 See Pence and Ramsey (2018) for a detailed version of this argument, and Mizrahi (2020) for an assessment of the case-study method in this respect. See Lean et al. (2021) for an account of how to bridge the gap between digital methods and practice-based philosophy of science. See, also, among others Malaterre and Chartier (2021); Noichl (2021); Malaterre et al. (2020); Herfeld and Doehne (2019); Böhm et al. (2022) for applications of computational methods in the philosophy of science. See also Sørensen and Johansen (2020) for a philosophical study of mathematical diagrams using machine-learning methods, whose outlook is particularly close to our present one.

10 Hobbes (1651, p. 55), see also Adams (2019).

11 d’Alambert (1821, p. 115).

12 Weingart (2013a) also provides a nice illustrative collection of these diagrams. For another great collection of science maps, see Börner (2010).

13 See Petrovich (2020) for a very good overview.

14 See Boyack et al. (2005), Klavans and Boyack (2017). See also van Eck and Waltman (2010) for an interactive interface for the construction and exploration of citation networks.

15 We should note here that the actual orientation of the mapping is an artifact of the algorithm, and has no meaning. Rotated, or mirrored mappings would be equivalent to the original while stretching or distorting the maps would not be permissible.

16 Compare Klavans and Boyack (2009).

17 See the results of Carlson and Harris (2020).

18 See Abdill and Blekhman (2019), Xie et al. (2021).

19 We thank Herold (2022) for their code and advice.

20 See Clement et al. (2019) for the introduction of the collection-mechanism.

21 The whole code of this analysis is made available in a GitHub repository under https://github.com/MNoichl/comp_templates. Similar analyses of textual data can found in Low et al. (2020), Javier et al. (2022), Ordun et al. (2020).

22 This makes sense for our task, as we are interested in thematic structures. If we were trying to measure stylistic differences between articles, we shouldn’t be as drastic here.

23 Using nltk’s (Bird et al. 2009) implementation of the word-net-lemmatizer.

24 Virtanen et al. (2020).

25 This part of the analysis, including the application of SVD, is conducted in the framework provided by scikit-learn, Pedregosa et al. (2011).

26 We use the list implemented in nltk Bird et al. (2009). For an overview of commonly used lists of stopwords, c.f. Nothman et al. (2018).

27 We must note here that UMAP is by no means the only available technique for this purpose. Further work might also consider the use of t-SNE, which, as González-Márquez et al. (2022) has recently argued, is well suited for the representation of text-corpora.

28 These problems have recently led to sharp critiques of the usage of these and similar techniques in the field of single cell-genomics (Chari et al. 2021), where they had become very established. As we do not use the coordinates acquired through UMAP for further analysis and remain firmly on the side of explorative data analysis, we believe to avoid the issues that linger here for the most part.

29 We here use the implementation of the Louvain-algorithm by Hollocou (2020).

30 As the BioRxiv archives formulas as images, we had to employ an online service, mathpix.com for conversion.

31 Joulin et al. (2016), Bojanowski et al. (2017).

32 For a philosophical localization of word-embeddings, see Gastaldi (2021).

33 This was an especially important feature of FastText compared to other models of the period, as it improved the quality of representations for languages that tend towards long word-combinations. The German ‘Aufmerksamkeitsdefizit-Hyperaktivitätsstörung’ (ADHD), might in itself be for example a very rare word, but it contains the n-grams from its constituting words, ‘Aufmerksamkeit’, ‘Defizit’, etc. from which its meaning can be (at least roughly) reconstituted.

34 The important point here is, that we can do this even for texts that have no specific words in common, as we have learned *semantic representations.*

35 McInnes et al. (2017).

36 A process not dissimilar to the one proposed by Ovchinnikova and Anders (2020).
